# The disease burden of childhood asthma in China: a systematic review and meta-analysis

**DOI:** 10.7189/jogh.10.01081

**Published:** 2020-06

**Authors:** Xue Li, Peige Song, Yongjian Zhu, Haohao Lei, Kit Yee Chan, Harry Campbell, Evropi Theodoratou, Igor Rudan, on behalf of Global Health Epidemiology Research Group (GHERG)

**Affiliations:** 1 Centre for Global Health Research, Usher Institute for Population Health Sciences and Informatics, University of Edinburgh, Edinburgh, UK; 2 Department of Epidemiology and Biostatistics, School of Public Health, Imperial College London, London, UK; 3 Department of Cardiology, Zhengzhou University, Zhengzhou, China; 4 Department of Social Science, University College London, London, UK; 5 Cancer Research UK Edinburgh Centre, Medical Research Council Institute of Genetics and Molecular Medicine, University of Edinburgh, Edinburgh, UK; * Joint first authors

## Abstract

**Background:**

In China, childhood asthma prevalence showed a remarkable increase in the past decades. An updated epidemiological assessment of childhood asthma in China with a focus on prevalence and time trends is required.

**Methods:**

We systematically searched three main Chinese databases and one English database to identify epidemiological studies of the prevalence of childhood asthma in China. Asthma cases were defined according to one of the five sets of Chinese diagnostic criteria which were established by the Children Respiratory Disease Group. We estimated age- and sex-specific prevalence of asthma using a multilevel mixed-effects logistic regression. We presented the time trends of asthma prevalence between 1990 and 2020 by age, sex and setting (urban *vs* rural), and also estimated the number of children affected by asthma in 2010.

**Results:**

In 1990, the prevalence of asthma ranged from 0.13% (95% confidence interval (CI) = 0.10-0.20) in rural girls aged 14 years to 1.34% (95% CI = 1.11-1.67) in urban boys aged five years. In 2010, the overall prevalence of asthma in Chinese children aged 0-14 years was 2.12% (95% CI = 1.83-2.51), corresponding to 5.16 million children living with asthma. Children aged 5-9 years were with the highest prevalence estimate of 2.65% (95% CI = 2.31-3.12) and those aged 10-14 years were with the lowest (1.48%, 95% CI = 1.26-1.78). In 2020, it is expected that this disparity will continue, with the prevalence of asthma being at the lowest level among rural girls aged 14 years (1.11%, 95% CI = 0.82-1.54) and at the highest level among urban boys aged four years (10.27%, 95% CI = 8.61-12.18). Over the 30 years (1990-2020), the prevalence of asthma in children aged 0-14 years has increased in both sexes and settings, which was consistently the lowest in rural girls and the highest in urban boys.

**Conclusions:**

This study shows that childhood asthma has been increasingly prevalent in China. Asthma is more frequent in boys and in rural areas. The detailed and systematic estimates of asthma prevalence in this study constitute the best currently available basis for policymaking, planning, and allocation of health and welfare resources related to the burden of childhood asthma in China.

Asthma is deﬁned as a chronic inﬂammatory disorder of airways and is associated with airway hyper-responsiveness that leads to attacks of wheezing, breathlessness, cough and/or chest tightness with varying severity and frequency [1]. Globally, asthma has been recognized one of the most costly respiratory disorders, which has caused substantial disability, impaired quality of life and deaths among children and young adults [2]. The Global Burden of Disease Study (GBD) in 2015 estimated that asthma accounts for 1.1% of the overall global estimates of disability-adjusted life years lost [3]. Approximate one third to half of children with moderate to severe asthma may persist to adulthood, which has exerted heavy burden on family and health care system [4]. Importantly, a substantial increase in the prevalence of childhood asthma has been reported across the world [5]. The most striking increase was observed in westernized countries, such as the United Kingdom, Australia, and Canada, which already had high rates of reported asthma, and a significant increase has also been noted in Asia and Eastern Europe which had a relative lower baseline prevalence [5]. With current rising trends, it is foreseeable that asthma will become a more serious public health problem and is of great concern among medical and health professionals.

In China, childhood asthma prevalence showed a remarkable increase in the past decades. The Chinese national cooperative group conducted three sample surveys in 1990, 2000 and 2010 to assess the prevalence of asthma in children aged 0-14 years. The first survey in 1990 reported the prevalence of asthma ranging from 0.09 to 2.60% in urban-resident children with a national prevalence of 0.91% [6]. In 2000, the second survey showed that the prevalence of asthma had increased to 0.52%-3.34%, with a national prevalence of 1.54% [7]. In 2010, the third repeated survey found that the prevalence was estimated to be 0.42%-5.37% with a national prevalence of 2.32% [8]. Since then, no recent official statistics have been made available on the current status of childhood asthma at the national level, though a multitude of new sub-national surveys have been conducted in different cities of China showing considerable regional variations [9-11].

Given the availability of national and sub-national investigations in childhood asthma in China, it is possible for us to provide an up-to-date analysis of the prevalence rate of asthma among Chinese children. In this study, we conducted a systematic review and meta-analysis to investigate the prevalence of asthma in Chinese children aged 0-14 years. The time trends (1990-2020) and variations of asthma prevalence by age, sex and setting (urban vs rural) were also explored. Moreover, the number of children affected by asthma in 2020 was also estimated.

## METHODS

### Systematic review and data extraction

This systematic review and meta-analysis was conducted in accordance with the Preferred Reporting Items for Systematic reviews and Meta-Analyses (PRISMA) statement [12]. Four bibliographic databases, including China National Knowledge Infrastructure (CNKI), Wanfang Data and VIP Database for Chinese Technical Periodical (VIP) and PubMed, were searched on 16th March 2019 to retrieve all epidemiological studies of asthma in Chinese children that were published from 1990 onwards. All titles, abstracts and keywords, followed by full texts of the records were examined by two independent trained reviewers (CNKI, Wanfang and VIP: XL, PS and HL; PubMed: XL and YZ) in parallel. Briefly, only studies that were conducted in the general paediatric population in Mainland China were included. The eligible studies should have reported the numerical prevalence of asthma in children, and asthma should be defined according to one of the five sets of Chinese diagnostic criteria which were established by the first Children Respiratory Disease Conference in 1987, the Nation Cooperation Group on Childhood Asthma (NCGCA) of 1993 or 1998, and the Branch for Respiratory Diseases of Chinese Medical Associations (BRDCMA) of 1997 or 2003 [13]. For multiple publications based on the same study, the one with the most detailed information was kept.

All relevant data, including author(s), year of publication, study design, sampling method, setting (urban, rural or mixed), year of investigation, asthma diagnosis, sample size and asthma cases, were extracted from the included studies. Wherever prevalence was split into different subgroups, ie, age-, sex-, setting-specific prevalence, data were tabulated for those subgroups accordingly.

### Statistical analysis

In the data extraction procedure, individual studies were used to provide multiple data points which contributed to the overall data set. To take into account the sample size and the availability of different data points from the same study, a multilevel mixed-effects logistic regression was adopted with a restricted cubic spline [14,15]. This was then used to model the asthma prevalence as a function of age. Based on a total of 752 data points, the age-, sex- and setting-specific prevalence of asthma in Chinese children was estimated for the years 1990, 2000, 2010 and 2020. We further estimated the corresponding 95% confidence intervals (CIs) by applying the semiparametric bootstrapping method [16]. The number of asthma cases in Chinese children aged 14 years and younger in 2010 was calculated by multiplying the paediatric population size in China, available from the United Nations Population Division (UNPD) and the China Census 2010. All the analyses were conducted in R v3.3.0 (https://www.r-project.org/).

## RESULTS

**Figure 1:**
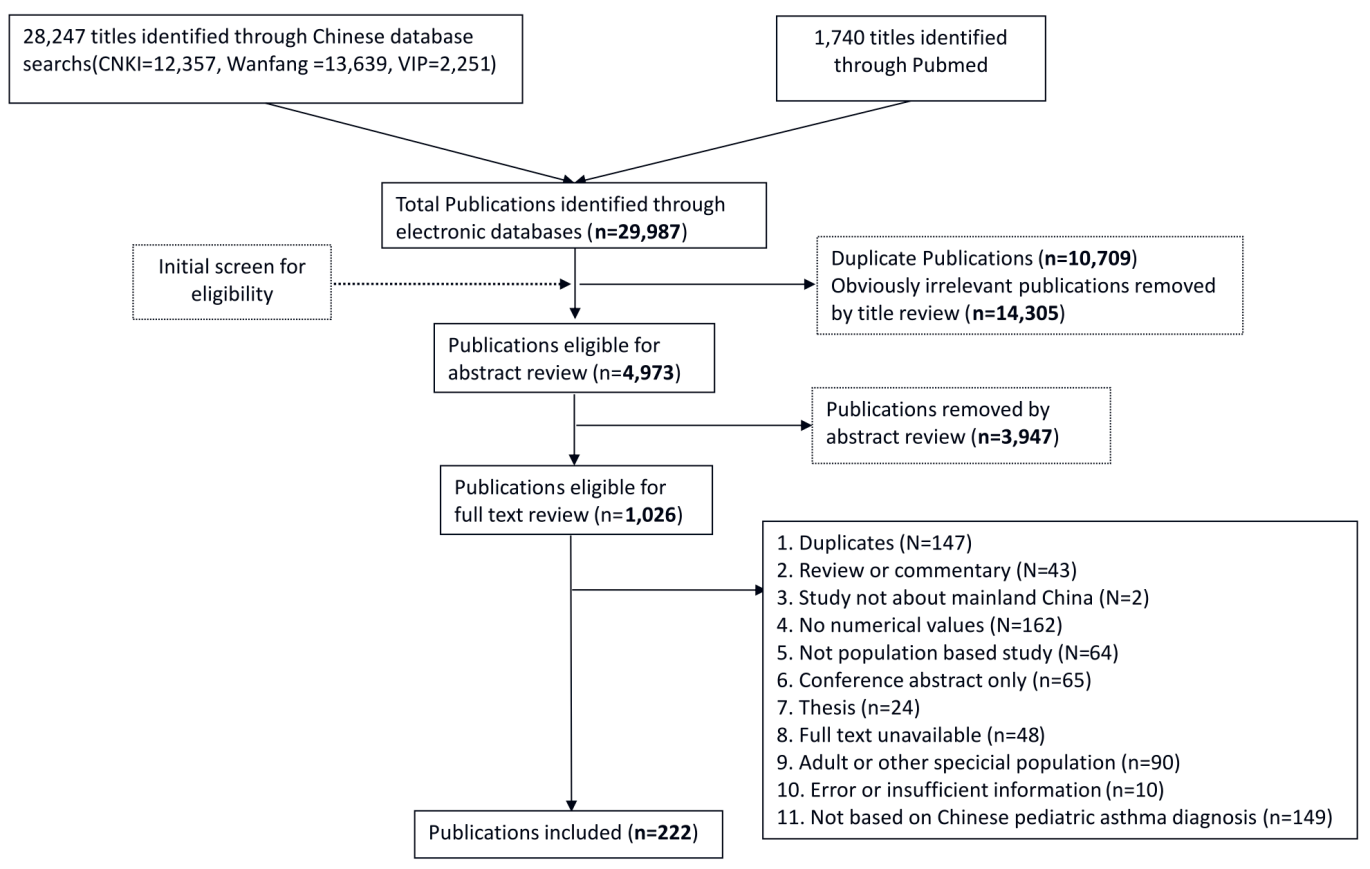
Flow diagram of systematic review process.

Our literature search returned a total of 29 987 citations. After removing 25 014 duplicates and irrelevant citations, 4973 records were screened, among which 1026 were reviewed at full-text. Finally, 222 articles were included according to the eligibility criteria. The process of study selection is documented in detail in **Figure 1****.** **Table 1** demonstrates the main characteristics of the remaining 222 articles, which were all cross-sectional in design and defined asthma according to one of the five Chinese diagnostic criteria. More than half of these articles were published in the most recent decade (n = 112, 50.5%) and most of the studies were conducted in urban areas (n = 142, 64.0%).

**Table 1:** Main characteristics of the included articles (n = 222)

Characteristic	Number of articles (%)
**Year published:**	
1990-1999	31 (14.0)
2000-2009	79 (35.6)
2010-2016	112 (50.5)
**Setting:**	
Urban	142 (64.0)
Rural	11 (5.0)
Mixed	69 (31.1)
Sample size:	
100-5000	30 (13.5)
5001–10 000	43 (19.4)
10 001–20 000	97 (43.7)
>20 000	52 (23.4)

**Figure 2:**
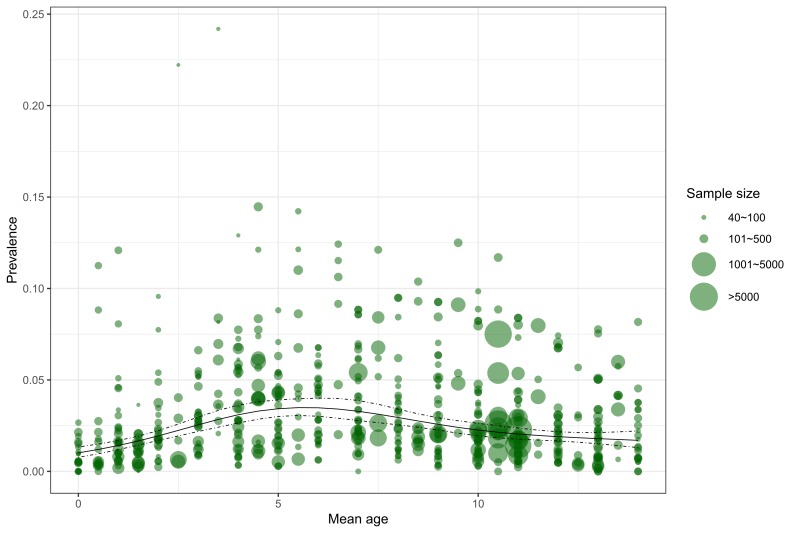
Age-specific prevalence of asthma in Chinese children based on the data points from the included studies. The spot sizes were scaled according to the study sample sizes.

A total of 55 retained articles provided the prevalence of asthma for different age and sex subgroups. Across the age span from zero to 14 years, the relation between age and asthma prevalence based on all informative data points is demonstrated in **Figure 2**. Generally, the prevalence of asthma steadily increased with age before five years old, and then started to decrease until 14 years old.

Age, sex, setting and study year were all significantly associated with asthma prevalence. The final formula generated from the multilevel mixed-effects logistic regression is shown below:

ln(odds) = –147.519 + 0.071 × *year*–0.503 × *sex_girl_* + 0.757 × *setting_urban_* + 0.300 × *age*1 – 0.939 × *age*2 + 1.959 × *age*3 – 1.074 × *age*4 + *ui*

Where

odds – p/(1 – p), p indicates the prevalence of asthma

year – calendar year

*sex_girl_* – 1 for girls and 0 for boys

*setting_urban_* – 1 for urban areas and 0 for rural areas

age1 – age4 are variables created in the process of fitting cubic splines (knots: 0.5, 4.0, 7.0, 10.0, and 13.5)

ui variance of the study-level random effect

**Figure 3:**
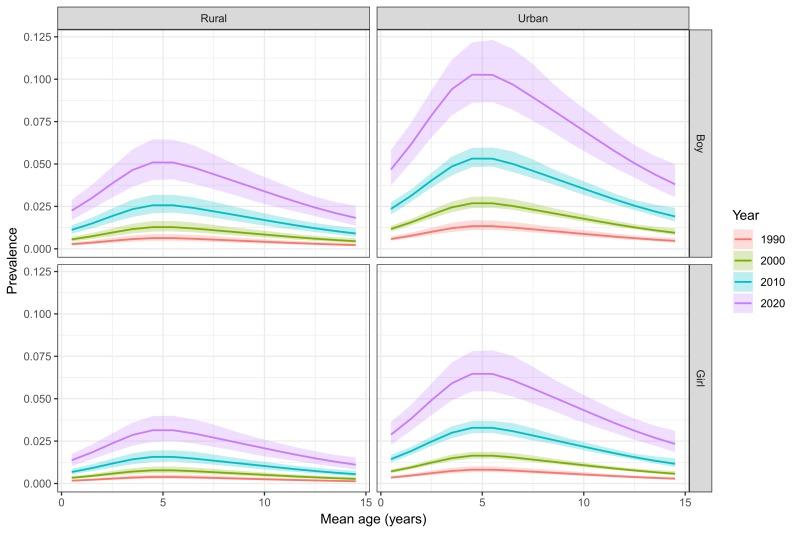
Age-, sex- and setting-specific prevalence of asthma in Chinese children in the years 1990, 2000, 2010 and 2020, with 95% confidence intervals.

Based on the final formula, age-specific prevalence of asthma in Chinese children was calculated for different sexes, settings and the years 1990, 2000, 2010 and 2020 respectively (**Figure 3** and Table S1 in the **Online Supplementary Document****)**. Over the 30 years (1990-2020), the prevalence of asthma in children aged 0-14 years has increased in both sexes and settings, which was consistently the lowest in rural girls and the highest in urban boys. In 1990, the prevalence of asthma ranged from 0.13% (95% CI = 0.10-0.20) in rural girls aged 14 years to 1.34% (95% CI = 1.11-1.67) in urban boys aged five years. In 2020, this disparity will continue, with the prevalence of asthma being at the lowest level among rural girls aged 14 years (1.11%, 95% CI = 0.82-1.54] and at the highest level among urban boys aged four years (10.27%, 95% CI = 8.61-12.18).

After applying the estimates of age-, sex- and setting-specific prevalence of asthma on United Nations Population Division's demographic data and China Census data, the overall prevalence of asthma and number of affected cases in Chinese children aged under 5 years (0-4 years), 5-9 years, 10-14 years and 0-14 years were calculated for the year 2010 (**Table 2** and Figure S1 in the **Online Supplementary Document**). Overall, the prevalence of asthma in Chinese children aged 0-14 years was 2.12% (95% CI = 1.83-2.51) in 2010. Children aged 5-9 years were with the highest prevalence estimate of 2.65% (95% CI = 2.31-3.12) and those aged 10-14 years were with the lowest (1.48%, 95% CI = 1.26-1.78). Regarding the number of asthma cases, the total number of affected children aged 0-14 years was 5.16 million (95% CI = 4.45-6.09) in China in 2010, among whom those aged 5-9 years contributed the most cases (40.5%, 2.09 million, 95% CI = 1.83-2.47). In 2010, more than one third (39.5%) of the paediatric asthma cases were boys living in urban areas.

**Table 2:** Estimated sex- and setting-specific prevalence of and numbers of children with asthma in China in 2010, by broad age group

Age group	Prevalence of asthma (%)					Children with asthma (millions)				
**Urban**		**Rural**		**Overall**	**Urban**		**Rural**		**Overall**
**Boy**	**Girl**	**Boy**	**Girl**	**Overall**	**Boy**	**Girl**	**Boy**	**Girl**	
0-4 years	3.99 (3.56-4.47)	2.23 (1.98-2.59)	1.46 (0.99-2.09)	0.88 (0.59-1.27)	2.26 (1.93-2.65)	0.72 (0.65-0.81)	0.38 (0.34-0.43)	0.50 (0.40-0.62)	0.26 (0.21-0.32)	1.87 (1.60-2.19)
5-9 years	4.57 (4.14-5.18)	2.93 (2.62-3.40)	1.95 (1.34-2.82)	1.17 (0.79-1.71)	2.65 (2.31-3.12)	0.83 (0.76-0.94)	0.45 (0.40-0.51)	0.53 (0.44-0.66)	0.28 (0.23-0.35)	2.09 (1.83-2.47)
10-14 years	2.57 (2.28-2.97)	1.63 (1.42-1.91)	1.08 (0.72-1.59)	0.64 (0.43-0.94)	1.48 (1.26-1.78)	0.49 (0.43-0.56)	0.26 (0.23-0.30)	0.29 (0.24-0.37)	0.16 (0.13-0.20)	1.20 (1.03-1.44)
0-14 years	3.69 (3.31-4.19)	2.25 (2.00-2.62)	1.49 (1.02-2.16)	0.89 (0.60-1.30)	2.12 (1.83-2.51)	2.04 (1.83-2.32)	1.09 (0.98-1.24)	1.33 (1.08-1.65)	0.70 (0.56-0.88)	5.16 (4.45-6.09)

## DISCUSSION

In this study, by systematically reviewing all published evidence of childhood asthma prevalence and applying strict inclusion and exclusion criteria, we present a comprehensive estimate of the pooled prevalence and epidemic characteristics of asthma in Chinese children, including its time trend, sex difference, age structures and the predicted prevalence in the upcoming year 2020. We identified 268 articles reporting the prevalence of childhood asthma, but only these (n = 222) defining asthma cases by following one of the five versions of Chinese diagnostic criteria were finally included for analysis. As these versions of Chinese criteria were all quite similar, therefore we did not consider that these minor difference would substantially distort the comparison of asthma prevalence between studies. Additionally, a small number of studies followed the protocol of The International Study of Asthma and Allergies in Childhood (ISAAC) and most of them were published prior to 2010 [5]. Studies based on ISAAC protocol relied on self-reported or parental reports of asthma [5], while the Chinese diagnostic criteria relied on the physician to evaluate the collected information of each asthma potential case and make sure that a set of necessary of criteria were fulfilled [13]. The differences described above point towards the need for reasonable similar disease definitions and data collection methods in order to be able to synthesize prevalence between studies. Given the sample size was large for articles based on Chinese diagnostic criteria, we therefore restrict our analysis to the Chinese diagnostic criteria only.

With following the Chinese diagnostic criteria, we estimated an overall prevalence of 2.12% for children aged 0-14 years, corresponding to 5.16 million children living with asthma in 2010. Our estimates were generally consistent with these reported in two published meta-analyses. In 2012, Yangzong *et al* reviewed the epidemiology studies of childhood asthma in China, and identified 12 studies followed the ISAAC protocol whereas 62 studies followed the Chinese diagnostic criteria [17]. They reported generally low prevalence following both criteria, whereas time trends in the prevalence were not summarized. Another meta-analysis of 117 studies that were published between 1988 and 2014 and followed the Chinese diagnostic criteria with a total sample size of 2 678 696 was conducted subsequently, and reported an overall asthma prevalence of 2.11% in children [18]. Their estimate is quite similar to ours. Our study observed an overall increasing trend of childhood asthma prevalence during the two past decades, from 0.91% in 1990, to 1.54% in 2000, to 2.12% in 2010, and indicated the prevalence of asthma in Chinese children having increased by almost 50% every 10 years. Our estimates were much in line with the increasing trend of asthma prevalence globally, though they are much lower than the current prevalence in many other countries, especially in developed countries, such as Austria (11.3%), United States (8.69%), United Kingdom (15.4%) and Canada (13%) [2,19]. When taking into account the facts that China possessed the highest asthma mortality rate (36.7 deaths per 100 000 patients with asthma) [2] and the second large population in the world, the disease burden attributed to asthma could be as heavy as these high prevalent countries.

Furthermore, the prevalence had more often been assessed in urban than in rural areas, making it difficult to some extent to compare the difference between urban and rural areas. Most of the surveys had been conducted in large and modern cities, like Shanghai and Beijing. With a wealth of data from urban areas, the prevalence of childhood asthma was higher than that in rural areas. In Beijing, the total prevalence of asthma in children aged below 14 years in rural areas (1.3%) was much lower than that in urban areas (3.7%) [9]. These findings are consistent with the idea that the degree of modernization or westernization is related to high prevalence of asthma [20]. In China, rural areas are generally with less or lower–quality health resources and worse health outcomes in comparison to urban areas [21,22]. This urban-rural disparity of asthma prevalence may be associated with prevalent indoor and outdoor air pollution resulting from multifarious interior decoration or heavy traffics in urban areas [23,24]. In addition, lower socioeconomic status and lower health resource quality in rural areas may also have contributed to the under-diagnosis of asthma [25].

As expected, asthma prevalence retains to be higher in boys than in girls (3.69% vs 2.25% in urban areas). When comparing to adults, the asthma prevalence is higher in women than in men, indicating a switch during puberty [26]. This pattern has raised several hypotheses to explain the sex difference, such as effects of sex hormones, airway calibre, and differences in exposure or diagnosis [27]. The higher prevalence in boys could be partly due to their smaller airway relative to lung size compared with young girls, and this patter reverses during adolescence [28]. Other possible explanations include hormonal changes and sex-specific differences in genetic susceptibility [29]. In particular, sex hormones are indicated to play an important role in the development and outcome of the allergic immune response, in particular asthma, as outlined by many studies [27,29,30]. A better understanding of precisely how sex hormones change the immune system’s response to allergens has the potential to help us tailor asthma therapy more specifically.

In view of the relation between asthma prevalence and age, our study confirms that children aged 5-9 years have the highest prevalence (2.65%). Children in this age group always have a weak immune system and low self-management abilities, thus they are vulnerable to the upper respiratory infection, which is thought to be one of the main contributors of childhood asthma [31]. A similar condition was observed in the meta-analysis reported by Guo et al [18]. Along with the increase of age, asthma prevalence decreases at 10-14 years. This decrease might be attributed to the strengthened self-management abilities and stronger immunity. Additionally, the reported prevalence for children under age five should be interpreted with caution. Special challenges in asthma diagnosis must be taken into account, as the diagnosis of asthma in younger children has to be largely based on clinical judgment and an assessment of symptoms and physical findings [32]. In this case, a confident diagnosis of asthma can be difficult because episodic respiratory symptoms such as wheezing and cough are also common in children without asthma in this age group. Importantly, it should be realised that reliable quantitative indicators are made available in the most recently updated guideline in 2016 [33] and a new scoring system is also recently initiated in China to establish a diagnostic system for asthma in young children and babies [34]. Promoting the physician’s knowledge and adherence to the updated Chinese guideline and recommendations would considerably improve the diagnosis and thus ensure an accurate estimate of the disease burden of asthma in younger children in China.

Our study also has several potential limitations. First, significant heterogeneity was seen between the included studies. This could be partly due to the regional difference, given that China is a diverse country with highly varied culture, levels of development and demographic characteristics. It could also be attributed to the increasingly recognized heterogeneity of asthma, as there is no gold-standard diagnostic test. Although only studies that adopted the Chinese diagnostic criteria were included, in order to reduce the methodological variability, heterogeneity may still exist because of the differences among investigators in operating protocols, levels of training, adherence to guidelines and variations in the implementation of guidelines. Moreover, there is increasing community awareness of asthma, leading to changing diagnostic habits and international classification had also changed over time, all leading to difficulties determining the reality and accuracy of reported trends. Another possible cause for concern is that the results of meta-regression only took into account a limited set of covariates (ie, sex, age, year and urbanicity) because these were the only covariates that were broadly available. The scarcity of studies adopting unified definitions of other covariates limited our ability to explore more possible effects.

## CONCLUSIONS

In conclusion, results from this study have shown that childhood asthma is increasingly prevalent in China. Asthma is more frequent in boys and in rural areas. Further analysis from an epidemiological perspective will remain to be required to explore the temporal distribution of asthma prevalence in Chinese children. The detailed and systematic estimates of asthma prevalence and the number of cases in this study constitute the best currently available basis for policymaking, planning, and allocation of health and welfare resources related to the burden of childhood asthma in China. Optimal intervention delivery - both in primary and secondary prevention and treatment - is urgently needed to counter this growing trend.

## Additional material

Online Supplementary Document
