# Supplementary material for: The disease burden of childhood asthma in China: a systematic review and meta-analysis
Source: J Glob Health. 2020 Feb 20;10(1):010801. doi: 10.7189/jogh.10.01081 (PMC7101212; doi:10.7189/jogh.10.01081)
Supplement: Online Supplementary Document [file jogh-10-010801-s001.pdf]

Table S1. Estimated age-, sex- and setting-specific prevalence of asthma in Chinese children in the years 1990, 2000, 2010 and 2020

Age	Urban							
	Boy				Girl			
	1990	2000	2010	2020	1990	2000	2010	2020
<1 year	0.58 (0.47-0.72)	1.17 (1.01-1.36)	2.35 (2.01-2.74)	4.67 (3.74-5.82)	0.35 (0.28-0.44)	0.71 (0.61-0.84)	1.43 (1.23-1.68)	2.88 (2.28-3.63)
1 year	0.77 (0.64-0.96)	1.56 (1.39-1.76)	3.13 (2.78-3.51)	6.18 (5.10-7.44)	0.47 (0.39-0.58)	0.95 (0.84-1.08)	1.92 (1.70-2.16)	3.83 (3.13-4.65)
2 years	1.00 (0.82-1.24)	2.02 (1.79-2.28)	4.03 (3.61-4.48)	7.88 (6.57-9.36)	0.61 (0.50-0.76)	1.23 (1.10-1.40)	2.48 (2.21-2.77)	4.91 (4.04-5.91)
3 years	1.22 (0.99-1.53)	2.45 (2.16-2.78)	4.86 (4.34-5.43)	9.41 (7.88-11.15)	0.74 (0.60-0.93)	1.49 (1.32-1.71)	2.99 (2.67-3.37)	5.91 (4.90-7.14)
4 years	1.34 (1.10-1.68)	2.69 (2.40-3.07)	5.32 (4.82-5.95)	10.27 (8.61-12.18)	0.81 (0.67-1.02)	1.64 (1.46-1.87)	3.28 (2.97-3.69)	6.47 (5.44-7.81)
5 years	1.34 (1.11-1.67)	2.68 (2.42-3.08)	5.32 (4.85-5.97)	10.26 (8.66-12.32)	0.81 (0.67-1.02)	1.64 (1.48-1.87)	3.28 (2.98-3.69)	6.46 (5.43-7.85)
6 years	1.26 (1.05-1.58)	2.52 (2.29-2.93)	5.01 (4.52-5.74)	9.69 (8.20-11.79)	0.76 (0.63-0.96)	1.54 (1.38-1.80)	3.09 (2.77-3.56)	6.09 (5.12-7.52)
7 years	1.15 (0.97-1.44)	2.31 (2.09-2.69)	4.59 (4.16-5.27)	8.93 (7.55-10.88)	0.70 (0.58-0.88)	1.41 (1.27-1.65)	2.83 (2.54-3.26)	5.59 (4.71-6.89)
8 years	1.04 (0.87-1.29)	2.01 (1.91-2.39)	4.18 (3.79-4.69)	8.15 (6.87-9.73)	0.63 (0.53-0.80)	1.28 (1.16-1.47)	2.57 (2.34-2.90)	5.09 (4.28-6.16)
9 years	0.93 (0.77-1.16)	1.88 (1.70-2.16)	3.75 (3.38-4.21)	7.36 (6.12-8.73)	0.57 (0.47-0.71)	1.14 (1.03-1.31)	2.30 (2.09-2.58)	4.58 (3.80-5.53)
10 years	0.82 (0.68-1.04)	1.67 (1.49-1.92)	3.33 (2.98-3.75)	6.56 (5.43-7.85)	0.50 (0.41-0.62)	1.01 (0.90-1.15)	2.04 (1.83-2.30)	4.07 (3.37-4.92)
11 years	0.72 (0.59-0.90)	1.46 (1.32-1.67)	2.92 (2.64-3.26)	5.78 (4.78-6.92)	0.44 (0.36-0.54)	0.89 (0.79-1.01)	1.79 (1.62-2.02)	3.57 (2.98-4.34)
12 years	0.63 (0.52-0.78)	1.27 (1.15-1.45)	2.54 (2.29-2.87)	5.04 (4.19-6.12)	0.38 (0.31-0.48)	0.77 (0.69-0.88)	1.55 (1.41-1.76)	3.11 (2.58-3.82)
13 years	0.54 (0.44-0.69)	1.09 (0.98-1.31)	2.20 (1.94-2.61)	4.38 (3.58-5.48)	0.33 (0.27-0.42)	0.66 (0.58-0.80)	1.34 (1.18-1.60)	2.70 (2.22-3.41)
14 years	0.47 (0.37-0.63)	0.95 (0.80-1.21)	1.91 (1.61-2.42)	3.80 (3.04-4.99)	0.28 (0.22-0.39)	0.57 (0.47-0.74)	1.16 (0.97-1.48)	2.33 (1.84-3.10)

Age	Rural							
	Boy				Girl			
	1990	2000	2010	2020	1990	2000	2010	2020
<1 year	0.27 (0.20-0.37)	0.55 (0.43-0.71)	1.12 (0.88-1.39)	2.25 (1.69-2.88)	0.16 (0.12-0.22)	0.33 (0.26-0.43)	0.68 (0.54-0.86)	1.37 (1.03-1.75)
1 year	0.36 (0.27-0.49)	0.74 (0.57-0.93)	1.49 (1.21-1.84)	3.00 (2.36-3.77)	0.22 (0.16-0.30)	0.45 (0.35-0.57)	0.91 (0.73-1.13)	1.83 (1.43-2.29)
2 years	0.47 (0.35-0.65)	0.96 (0.74-1.21)	1.93 (1.56-2.38)	3.86 (3.02-4.83)	0.29 (0.21-0.39)	0.58 (0.45-0.75)	1.18 (0.93-1.47)	2.37 (1.83-2.95)
3 years	0.57 (0.42-0.79)	1.16 (0.91-1.48)	2.34 (1.88-2.90)	4.65 (3.66-5.87)	0.35 (0.25-0.48)	0.71 (0.55-0.91)	1.43 (1.13-1.78)	2.86 (2.22-3.60)
4 years	0.63 (0.47-0.87)	1.28 (1.01-1.64)	2.57 (2.10-3.18)	5.09 (4.06-6.46)	0.38 (0.28-0.53)	0.78 (0.61-1.00)	1.57 (1.27-1.95)	3.14 (2.46-3.97)
5 years	0.63 (0.47-0.87)	1.28 (1.03-1.64)	2.57 (2.13-3.19)	5.09 (4.09-6.43)	0.38 (0.28-0.53)	0.78 (0.62-1.00)	1.57 (1.29-1.96)	3.14 (2.51-4.00)
6 years	0.59 (0.44-0.83)	1.20 (0.97-1.55)	2.41 (2.01-3.02)	4.79 (3.84-6.11)	0.36 (0.27-0.51)	0.73 (0.59-0.95)	1.47 (1.21-1.87)	2.95 (2.36-3.78)
7 years	0.54 (0.40-0.76)	1.10 (0.88-1.41)	2.21 (1.83-2.78)	4.40 (3.52-5.57)	0.33 (0.25-0.46)	0.67 (0.53-0.86)	1.35 (1.11-1.71)	2.71 (2.16-3.47)

8 years	0.49 (0.37-0.68)	0.99 (0.79-1.26)	2.00 (1.65-2.49)	4.00 (3.18-5.01)	0.30 (0.22-0.41)	0.60 (0.48-0.77)	1.22 (1.00-1.52)	2.45 (1.95-3.09)
9 years	0.44 (0.33-0.60)	0.89 (0.71-1.13)	1.80 (1.45-2.22)	3.59 (2.81-4.48)	0.27 (0.19-0.37)	0.54 (0.43-0.69)	1.09 (0.88-1.36)	2.20 (1.71-2.74)
10 years	0.39 (0.28-0.53)	0.79 (0.62-1.00)	1.59 (1.28-1.96)	3.19 (2.50-4.00)	0.24 (0.17-0.33)	0.48 (0.37-0.61)	0.97 (0.77-1.20)	1.95 (1.50-2.44)
11 years	0.34 (0.25-0.46)	0.69 (0.54-0.87)	1.39 (1.13-1.73)	2.80 (2.20-3.51)	0.21 (0.15-0.28)	0.42 (0.32-0.54)	0.85 (0.68-1.05)	1.71 (1.33-2.15)
12 years	0.29 (0.22-0.40)	0.60 (0.47-0.77)	1.21 (0.98-1.52)	2.43 (1.92-3.07)	0.18 (0.13-0.25)	0.36 (0.29-0.47)	0.73 (0.60-0.93)	1.48 (1.15-1.90)
13 years	0.25 (0.19-0.36)	0.52 (0.41-0.69)	1.05 (0.85-1.36)	2.11 (1.65-2.76)	0.15 (0.11-0.22)	0.31 (0.24-0.42)	0.63 (0.51-0.83)	1.28 (0.98-1.69)
14 years	0.22 (0.16-0.33)	0.45 (0.35-0.63)	0.90 (0.70-1.22)	1.82 (1.37-2.53)	0.13 (0.10-0.20)	0.27 (0.21-0.38)	0.55 (0.43-0.75)	1.11 (0.82-1.54)

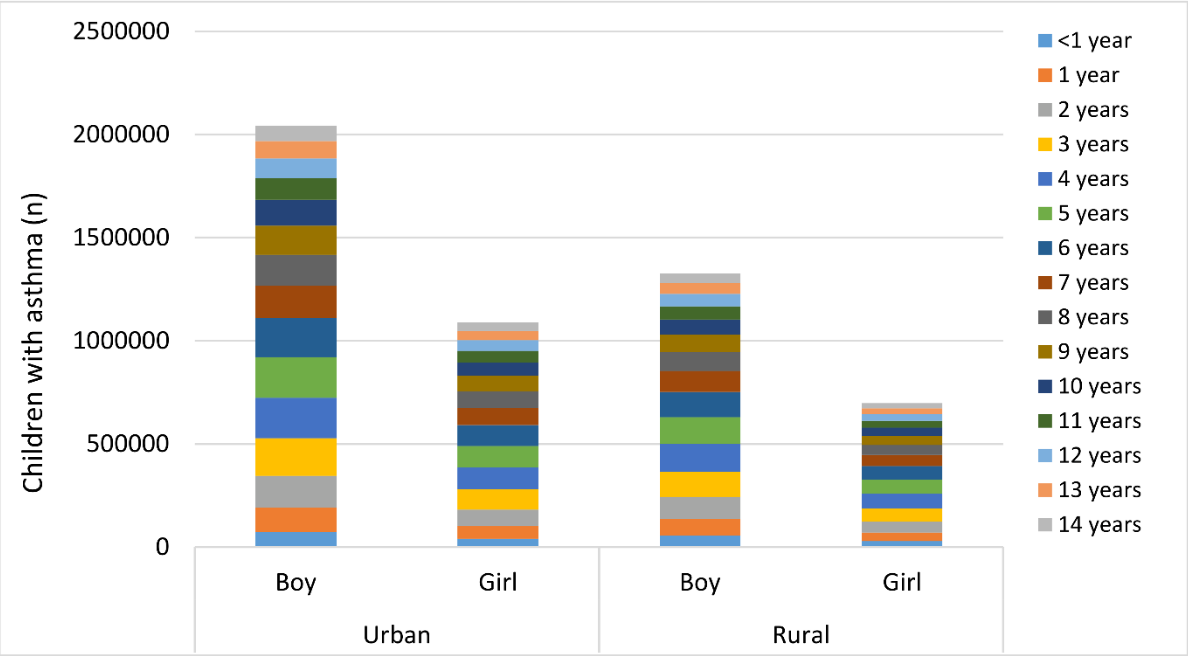

Figure S1. Estimated numbers of children with asthma in China by sex, setting and age in 2010.
